# Tumor-Associated Macrophages in Pancreatic Ductal Adenocarcinoma: Therapeutic Opportunities and Clinical Challenges

**DOI:** 10.3390/cancers13122860

**Published:** 2021-06-08

**Authors:** Ashleigh R. Poh, Matthias Ernst

**Affiliations:** Olivia Newton-John Cancer Research Institute and La Trobe University School of Cancer Medicine, Heidelberg, VIC 3084, Australia; Matthias.Ernst@onjcri.org.au

**Keywords:** pancreatic ductal adenocarcinoma, tumor microenvironment, macrophages, tumor immunology, immunotherapy

## Abstract

**Simple Summary:**

Macrophages are a major component of the pancreatic tumor microenvironment, and their increased abundance is associated with poor patient survival. Given the multi-faceted role of macrophages in promoting pancreatic tumor development and progression, these cells represent promising targets for anti-cancer therapy.

**Abstract:**

Pancreatic ductal adenocarcinoma (PDAC) is an aggressive malignant disease with a 5-year survival rate of less than 10%. Macrophages are one of the earliest infiltrating cells in the pancreatic tumor microenvironment, and are associated with an increased risk of disease progression, recurrence, metastasis, and shorter overall survival. Pre-clinical studies have demonstrated an unequivocal role of macrophages in PDAC by contributing to chronic inflammation, cancer cell stemness, desmoplasia, immune suppression, angiogenesis, invasion, metastasis, and drug resistance. Several macrophage-targeting therapies have also been investigated in pre-clinical models, and include macrophage depletion, inhibiting macrophage recruitment, and macrophage reprogramming. However, the effectiveness of these drugs in pre-clinical models has not always translated into clinical trials. In this review, we discuss the molecular mechanisms that underpin macrophage heterogeneity within the pancreatic tumor microenvironment, and examine the contribution of macrophages at various stages of PDAC progression. We also provide a comprehensive update of macrophage-targeting therapies that are currently undergoing clinical evaluation, and discuss clinical challenges associated with these treatment modalities in human PDAC patients.

## 1. Introduction

Pancreatic ductal adenocarcinoma (PDAC) is an aggressive malignant disease with a 5-year survival rate of less than 10% and is the seventh leading cause of cancer-related death worldwide [1,2]. In contrast to other cancer types, the survival rate of PDAC patients has not improved substantially over the past 40 years [3,4]. Two chemotherapy combinations, Gemcitabine/nab-Paclitaxel and FOLFIRINOX (irinotecan, oxaliplatin, 5-fluororacil and leucovorin), are currently used to treat PDAC; however, response to either of these two regimens is only observed in up to 30% of patients [4,5,6]. While surgery offers a potential cure, less than 20% of cases are resectable at diagnosis and most patients that undergo surgery still die of the disease due to local recurrence and/or metastasis [7,8,9]. Thus, there is an unmet clinical need to improve survival outcomes for PDAC patients. 

A hallmark of PDAC is the presence of a dense desmoplastic (fibrotic) reaction that surrounds cancer cells and can account for up to 80% of the tumor mass [10]. It is comprised of a heterogeneous population of cells (e.g., fibroblasts, stellate cells, immune cells, endothelial cells), acellular components (e.g., fibrin, collagen, hyaluronic acid, fibronectin, growth factors, cytokines), and is characterized by biophysical features (e.g., low pH, hypoxia, high interstitial pressure) that interact to promote tumor growth and limit therapeutic response [11]. For example, the presence of a dense extracellular matrix and high interstitial fluid pressure within the stroma limits the delivery and distribution of anti-cancer drugs [12]. Meanwhile, immunosuppressive factors secreted by cancer-associated fibroblasts and myeloid cells enables immune escape by preventing the infiltration and activation of cytotoxic effector cells [13]. Due to the functional complexity of the PDAC tumor microenvironment, complementary stromal- and immune-targeted treatment strategies that can deconstruct the desmoplastic stroma and reduce immunosuppression may offer a rational treatment approach to improve therapy response in PDAC. 

Tumor-associated macrophages (TAMs) are one of the earliest infiltrating cells in pancreatic intraepithelial neoplasms and continue to increase during progression to invasive cancer [14,15]. Macrophage density is an independent prognostic factor in human PDAC patients and is associated with a higher risk of disease progression, recurrence, metastasis, and shorter overall survival [16]. Meanwhile, pre-clinical PDAC mouse models have provided unequivocal evidence for the importance of these cells in driving angiogenesis, matrix remodeling, immunosuppression, tumor cell invasion, and drug resistance [17,18,19,20,21]. In this review, we will delineate the contribution of TAMs in PDAC, and explore how they may be utilized as therapeutic targets. 

## 2. Origin of Macrophages in PDAC

**Figure 1:**
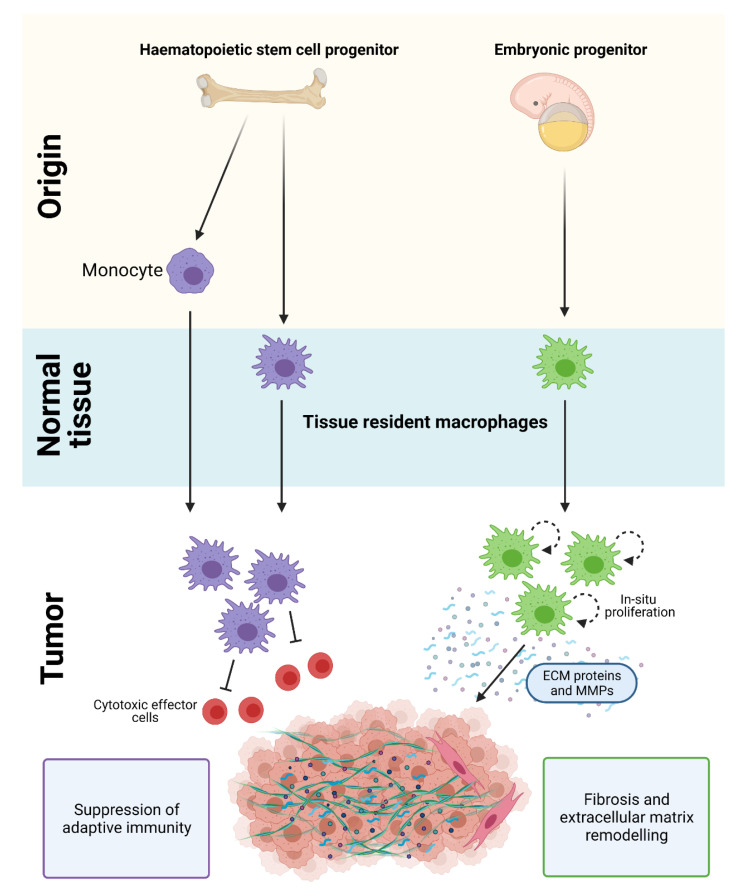
Origin of macrophages in PDAC. Macrophages in PDAC are derived from a mixed population of tissue-resident cells and circulating monocytes. HSC/monocyte-derived macrophages play a key role in regulating immune suppression and adaptive immunity, while embryonically-derived macrophages are important in promoting fibrosis and extracellular matrix remodeling in PDAC. Figure created in Biorender.

Macrophages in PDAC are derived from a mixed population of tissue-resident cells and circulating monocytes. Tissue-resident macrophages are diverse in their ontogeny and arise from embryonic precursors or from adult hematopoietic stem cell (HSC) progenitors. Monocyte-derived cells also contribute to the macrophage population in PDAC and are recruited into tissues by chemotactic signals [17,21,22,23] (Figure 1). Strikingly, loss of monocyte-derived macrophages has limited effects on PDAC progression, while depletion of tissue-resident macrophages significantly impairs tumor growth [17]. These results suggest that tissue-resident macrophages are more potent drivers of PDAC compared to their monocyte-derived counterparts, and are indispensable for tumor development.

Although the gene expression profile of embryonic- and HSC-derived macrophages are similar under normal homeostatic conditions, they exhibit distinct transcriptional profiles and ex vivo functions in PDAC (Figure 1). During tumor development, embryonically-derived macrophages undergo significant expansion in situ and proliferate at higher rates compared to their HSC-derived counterparts [17]. They also exhibit a pro-fibrotic transcriptional profile and produce significantly more collagen compared to HSC-derived TAMs, which suggests their involvement in ‘fine-tuning’ fibrotic responses [17,21,22,23]. In contrast, HSC/monocyte-derived macrophages have well-recognized roles in antigen presentation, immunosuppression, and therapeutic resistance [17,18,19,20,21]. While these findings suggest that embryonic- and HSC/monocyte-derived macrophages have distinct and non-redundant roles, other subsets derived from these two tissue origins are expected given the importance of microenvironmental cues in shaping macrophage plasticity. 

## 3. Macrophage Polarization

In addition to cellular ontogeny, macrophage heterogeneity is also influenced by niche-specific signaling events that occur within the tumor microenvironment. Macrophages are able to reversibly alter their phenotype in response to environmental cues, which include stimuli derived from pathogens, stromal and immune cells, as well as the extracellular matrix and metabolites [24]. In turn, this enables macrophages to perform a variety of different activities, including host defense, clearance of cellular debris, stimulating adaptive immunity, and wound healing. 

**Figure 2:**
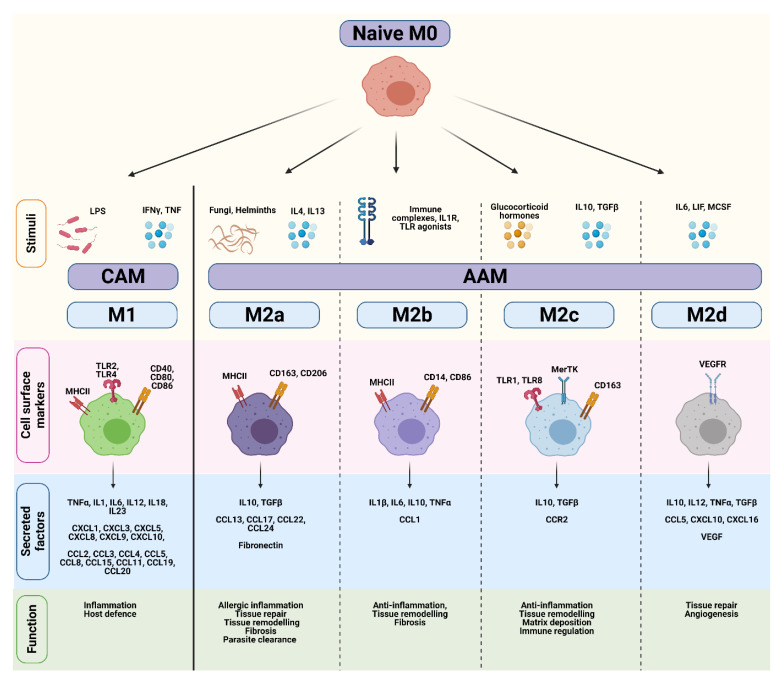
Comparison of CAM and AAM macrophage endotypes. Naïve M0 macrophages differentiate into classically-activated M1 (CAM) or alternatively-activated M2 (AAM) subtypes in response to various stimuli. CAMs and AAMs exhibit distinct surface markers, cytokines, and biological functions. Figure created in Biorender.

Most of our knowledge on macrophage polarization has relied on in vitro techniques, including the stimulation of naïve M0 macrophages with polarizing cytokines. Differences in transcriptional profiles, cell-surface markers, signaling pathways and biological functions have subsequently been used to distinguish various activation states, which are broadly divided into either ‘classically-activated M1’ (CAM) or ‘alternatively-activated M2’ (AAM) subtypes [24,25,26,27] (Figure 2). However, while the ‘M0 to M1/M2’ classification system represents a useful and simplified conceptual framework, this linear model does not reflect the true complexity of macrophage polarization in an in vivo tumor setting. Instead, macrophage polarization should be viewed as a highly dynamic and reversible process where TAMs exist on continuum of diverse functional states, and can encompass features of both ‘M1/M2’ phenotypes.

Classically-activated M1 macrophages are induced in response to bacterial lipopolysaccharide (LPS), or the Th1 cytokines IFNγ and TNFα (Figure 2) [26,28]. IFNγ and TNFα are produced by antigen-presenting cells upon recognition of pathogenic signals, but can also be secreted by T-cells and natural killer (NK) cells. Once activated, CAMs secrete pro-inflammatory cytokines (e.g., TNFα, IL1β, IL12), effector molecules (e.g., reactive nitrogen intermediates), and chemokines (e.g., CXCL9, CXCL10) to amplify and sustain their anti-microbial and tumoricidal activity [24]. Although these cytokines play an important role in host defense, sustained production of these molecules may result in chronic inflammation. A fine-tuned balance is therefore necessary to maintain homeostasis and prevent extensive tissue damage.

Alternatively-activated M2 macrophages play a pivotal role in wound healing and suppression/regulation of inflammatory responses. They are divided into at least four distinct subtypes (M2a, M2b, M2c, and M2d) based on their functional differences in response to different stimuli [25,29] (Figure 2). M2a macrophages are induced by IL4, IL13, and helminth and parasitic infections. They secrete high levels of immunosuppressive cytokines (e.g., IL10, TGFβ), chemokines (e.g., CCL13, CCL17), and components of the extracellular matrix to promote tissue repair, remodeling, and fibrosis. Common markers used to identify M2a macrophages include mannose receptor (CD206) and scavenger receptors such as CD163 [30]. M2b macrophages are induced by immune complexes and TLR agonists, and regulate immune responses through the production of pro-inflammatory (e.g., IL1β, IL6, TNFα) and anti-inflammatory cytokines (e.g., IL10) [31]. M2c macrophages are induced following stimulation with IL10, TGFβ, and glucocorticoids, and highly express Mer receptor tyrosine kinase (MerTK) which enables efficient phagocytosis of apoptotic cells [32]. Lastly, M2d macrophages are induced by IL6, and mediate tissue repair and angiogenesis through secretion of IL10 and VEGF [33,34]. Thus, AAMs encompass a functionally diverse group of cells that contribute to immunosuppression, tissue remodeling, and angiogenesis. 

## 4. Role of TAMs in PDAC

Although macrophages play a central role in host defense, inappropriate or prolonged activation can result in immune dysregulation, tissue damage, and disease [35]. Depending on their polarization, macrophages can play contrasting roles in tumor development and progression. For example, newly tumor-infiltrated naïve M0 macrophages exert anti-tumorigenic activities via TNFα secretion [36]. However, their subsequent differentiation into AAMs reduces TNFα levels and suppresses their cytotoxic activity [36]. 

**Figure 3:**
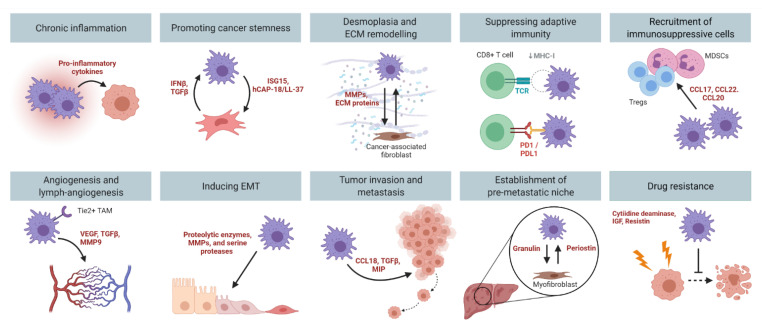
The multi-faceted role of TAMs in PDAC. Macrophages promote PDAC development and progression by contributing to chronic inflammation, promoting cancer stemness, desmoplasia, immune suppression, angiogenesis, inducing EMT, tumor invasion and metastasis, and mediating drug resistance. Figure created in Biorender.

Pre-clinical PDAC mouse models have provided unequivocal evidence for the importance of TAMs in tumor development and progression. Genetic ablation of macrophages blocks the progression of precancerous lesions to adenocarcinomas, and reduces tumor formation in mice [37,38]. Meanwhile, pharmacological depletion of TAMs impairs angiogenesis [39,40], tumor cell invasion [41,42,43,44], and metastasis [20,39]. These findings suggest that there is a fine-tuned balance between the tumoricidal and tumor-promoting functions of TAMs. To date, the tumor-promoting roles of macrophages in PDAC that have been well characterized include chronic inflammation, promoting cancer stemness, desmoplasia, immune suppression, angiogenesis, invasion, metastasis, and drug resistance (Figure 3).

### 4.1. Inflammation and Cancer Initiation

Chronic inflammation is a key mediator of early PDAC development, and promotes malignant progression and metastatic spread to distant organs [45,46]. Risk factors for PDAC that promote systemic inflammation include chronic pancreatitis [47,48], obesity [49], tobacco smoking [50], and alcohol use [51]. During acute pancreatitis, macrophages are predominantly skewed towards a classically-activated endotype that produce high levels of TNFα and IL1β [52,53,54]. In contrast, AAMs are the dominant subtype observed in chronic pancreatitis, and interact with pancreatic stellate cells to accelerate fibrosis [55].

In response to chronic inflammation, acinar pancreatic cells can adopt a metaplastic duct-like phenotype in a process known as acinar-to-ductal metaplasia (ADM) [56]. These precancerous lesions are frequently observed in pancreatitis [57], and may develop into pancreatic intraepithelial neoplasia (PanIN) following the acquisition of additional mutations such as oncogenic KRas [56,58]. Both ADM and PanIN are indispensable events in PDAC, and are marked by the progressive infiltration of macrophages that persist throughout tumor development [37,59,60]. Accordingly, macrophage depletion blocks the progression of ADM to PanIN, and reduces PDAC formation in mice [37,38].

Classically-activated M1 macrophages initiate ADM via the secretion of inflammatory mediators, including IL6 [61], TNFα, and RANTES [37,62]. IL6 contributes to ADM through activation of JAK-STAT3 signaling [61], while TNFα and RANTES activate NFκB in acinar cells to drive proliferation, survival, and extracellular matrix degradation [37,62]. In turn, acinar cells express intercellular adhesion molecule-1 (ICAM-1), which induces a paracrine loop that sustains local inflammation and ADM transformation [38]. TAMs can also enhance the expression of heparin-binding epidermal growth factor-like growth factor (EGFR ligand) in pre-neoplastic lesions to facilitate ADM and the development of PanIN lesions [63]. Once ADM progresses to PanIN, inflammatory macrophages are reprogrammed towards an alternatively-activated endotype in response to IL13 secreted by neoplastic cells, and drive fibrosis via IL1Ra and CCR2 signaling [64]. 

Although additional inflammatory insults and genetic alterations are needed to drive the progression of PanIN to PDAC [65,66], neoplastic cells from PanIN lesions can undergo epithelial-to-mesenchymal transition (EMT), circulate in the blood, and seed in the liver before primary tumor development [67,68]. EMT occurs when tumor cells lose their intrinsic polarity and instead acquire a motile mesenchymal cell phenotype [69]. Features of EMT and epithelial cell invasion are most abundant at inflammatory foci of PanIN, and circulating pancreatic cells can be detected during pancreatitis [67]. These data suggest that establishment of a metastatic niche may precede primary tumor development. Thus, inflammation caused by macrophages can promote PDAC progression by facilitating changes within the microenvironment at the primary site of neoplasia, and by enabling the invasion and dissemination of tumor cells into secondary organs.

### 4.2. Promoting Cancer Cell Stemness

Pancreatic cancer stem cells (PCSCs) are a rare population of immortal tumor cells that can self-renew and generate a heterogeneous lineage of cancer cells. Thus, PCSCs represent a cell population uniquely responsible for tumor heterogeneity, and play a fundamental role in tumor development, progression, and chemoresistance [70,71]. Although PCSCs can remodel their surroundings to maintain their self-renewing and tumorigenic properties, the tumor microenvironment also provides cues to upregulate EMT and stemness in PCSCs. Indeed, TAMs provide pivotal signals to promote PCSC survival, and in turn, PCSCs deliver tumor-promoting cues to TAMs to further enhance tumorigenesis. 

PCSC density positively correlates with abundance of TAMs in PDAC, and is associated with a reduced overall patient survival [72]. Targeting macrophage recruitment by blocking CCR2 or CSF1R signaling decreases the number of PCSCs, and improves response to chemotherapy [20]. TAMs directly enhance the tumor-initiating capacity of PCSCs by activating the transcription factor STAT3 in PCSCs, which facilitates macrophage-mediated suppression of CD8 T-cells [20]. Human PCSCs also secrete IFNβ to stimulate TAMs to produce the IFN-stimulated factor ISG15. Consequently, TAM-derived ISG15 reinforces the self-renewing, invasive capacity, and tumorigenic potential of PCSCs [73]. PCSCs are also a major source of the TGFβ superfamily members Nodal/Activin A and TGF*β*1, which induce the polarization of AAMs. In turn, AAMs secrete the antimicrobial peptide hCAP-18/LL-37, which binds to receptors on the surface of PCSCs to enhance PCSC self-renewal, invasion, and tumorigenesis [74]. Together, these studies suggest that the intricate cross-talk between PCSCs and TAMs is an important driver for tumor development in PDAC. 

### 4.3. Desmoplasia and ECM Remodeling

Desmoplasia is a prominent pathological characteristic of PDAC, and acts as a biophysical alteration that promotes tumor development, angiogenesis, metastasis, and drug resistance [11]. The dense and fibrous desmoplastic tissue is comprised of both cellular (e.g., fibroblasts, immune cells) and non-cellular connective tissue components (e.g., collagen, fibronectin) [11]. 

Pancreatic stellate cells (PSCs) are resident cells that maintain normal tissue architecture through the synthesis of ECM proteins and matrix-degrading enzymes such as matrix metalloproteinases (MMPs) [75]. In response to pancreatic injury or chronic inflammation, PSCs transform into an active myofibroblast-like phenotype and express high levels of alpha-smooth muscle actin [76,77]. This transition is accompanied by (1) an increase in proliferation and migration, (2) excessive synthesis of ECM proteins, MMPs and their inhibitors, and (3) the secretion of growth factors and cytokines that act in an autocrine and paracrine manner to enhance early tumor formation [75,76,77,78,79,80]. 

Reciprocal interactions between cancer-associated fibroblasts and AAMs trigger and sustain the desmoplastic reaction in PDAC. Co-culture of monocytes with PSCs and fibroblasts induces the production of immunosuppressive cytokines (e.g., IL6, IL8, IL10, IL33, GM-CSF, M-CSF), which promote the polarization of AAMs [81,82,83]. In turn, AAMs secrete growth factors (e.g., TGFβ1, PDGF, FGF2, granulin), cytokines (e.g., TNFα, IL1, IL6), and chemokines (e.g., CCL2) that directly activate fibroblasts and perpetuate a feedforward loop to sustain the desmoplastic reaction [84,85]. PI3Kγ signaling in macrophages also regulates the expression of platelet-derived growth factor-BB, which stimulates tumor cell chemotaxis and fibroblast production of collagen in vitro and in vivo [86]. Meanwhile, TAMs accumulate in hypoxic areas in response to HIF1α, and activate PSCs via CCL2 secretion [87]. Macrophages can also directly deposit and regulate the extracellular matrix via production of ECM-producing enzymes (e.g., hyaluronan synthases), and ECM-remodeling molecules (e.g., lysyl oxidase, MMPs) [17,55]. Thus, TAMs play an integral role in PDAC desmoplasia both directly and indirectly through their interactions with PSCs and cancer-associated fibroblasts. 

### 4.4. Immune Suppression

Macrophages are critical regulators of T-cell immunosurveillance, and establish a site of immune privilege in PDAC by inhibiting the recruitment of T-cells into the tumors [23]. TAMs also suppress T-cell activation via several major mechanisms, including (1) depletion of metabolites required for T-cell proliferation, (2) inhibition of T-cell activation by production of anti-inflammatory factors, (3) upregulation of inhibitory receptors to block T-cell function, and (4) generation of reactive oxygen species. Metabolism of L-arginine and L-tryptophan by TAMs decreases the expression of the CD3**ζ** chain on T-cells, and results in T-cell anergy and impaired proliferation [88,89,90]. Likewise, increased Arginase I production by AAMs interferes with T-cell metabolism and cytotoxicity [91].

TAMs also secrete anti-inflammatory/immunosuppressive cytokines (e.g., TGFβ, PGE2) and chemokines (e.g., CCL7, CCL2), which promote T-cell exhaustion and establishes a self-propagating tumor-permissive microenvironment [92,93,94,95]. In addition, macrophage-derived IL10 impairs NK cell proliferation [96,97], while TGFβ signaling in TAMs inhibits the cytolytic activity of NK cells in a contact-dependent manner [98].

TAMs can also upregulate the expression of ligands that bind to inhibitory receptors on the surface of T-cells to inhibit their activation, proliferation, and effector functions [99,100]. These include PDL1/PDL2 and CD80/CD86, which bind to PD1 and CTLA4, respectively on the surface of CD8 T-cells. Other ligands include Dectin1, which is highly expressed by macrophages in mouse and human PDAC [101]. Binding of Dectin1 to Galectin-9 on the surface of infiltrating immune cells and cancer cells results in tolerogenic macrophage programming and suppression of adaptive immune responses [101]. 

Numerous studies have established a role of TAMs in driving the polarization and expansion of immunosuppressive cell types into the PDAC microenvironment. NLRP3 signaling in macrophages drives the differentiation of CD4 T-cells into tumor-promoting Th2 cells, Th17 cells, and Tregs, while suppressing Th1 cell polarization and cytotoxic CD8 T-cell activation [102]. Macrophage-derived chemokines such as CCL17, CCL22, and CCL20 also induce the migration of Tregs into tumors [95], and promote the regulatory functions of these cells via IL10 and TGFβ signaling [103]. Thus, TAMs can suppress anti-tumor responses by preventing the recruitment and activation of cytotoxic effector cells, and by promoting the accumulation of Tregs into tumors. 

### 4.5. Angiogenesis and Lymph-Angiogenesis

Tumors require angiogenesis to obtain nutritional support and oxygen. TAMs are highly abundant in hypoxic areas of PDAC, and are associated with increased blood vessel formation via production of VEGF [104,105,106,107,108]. Upregulation of VEGF expression in TAMs requires the oncogenic transcription factors HIF1α, NFκB, and STAT3, which promote an angiogenic switch that enhances blood vessel formation and tumor growth [39]. In addition to their accumulation in primary tumors, VEGF-expressing TAMs are found in pre-metastatic niches where they promote tumor cell extravasation and vascular permeability [39]. Accordingly, macrophage depletion significantly impairs angiogenesis and reduces circulating levels of VEGF [39,40]. Macrophage-derived exosomes miR-155-5p and miR-221-5p can also promote angiogenesis in PDAC by suppressing expression of the transcription factor E2F2 in endothelial cells [108]. Moreover, pancreatic cancer cells produce cytokines such as IL35 to stimulate the angiogenic activity of TAMs via expression of CXCL1 and CXCL8, while inhibition of this interaction reduces TAM infiltration and microvessel formation [109]. A novel subset of TAMs expressing the tyrosine-protein kinase receptor TIE2 also exhibit enhanced pro-angiogenic activity in PDAC [110] via the binding of its receptor to angiopoietins to promote blood vessel formation [111,112]. Indeed, the abundance of TIE2 macrophages positively correlates with increased microvessel density and a greater risk of developing metastatic disease in PDAC [110].

TAMs also contribute to lymph-angiogenesis, which represents an important route of tumor cell dissemination to regional lymph nodes. In PDAC patients, high lymphatic density positively correlates with increased lymph node metastasis, and reduced overall patient survival [113,114]. Lymph-angiogenesis is dependent on the binding of VEGF-C (a ligand overexpressed by cancer cells) to its receptor VEGFR-3 (expressed on TAMs). The VEGF-C/VEGFR-3 axis can promote lymph-angiogenesis directly by activating lymphatic endothelial cells [115,116], or indirectly by increasing the secretion of molecules that induce lymph-angiogenesis including VEGF and MMP9 [117,118,119]. Of note, the presence of TAMs at the invasive front of PDAC tumors is associated with a poor prognosis due to accelerated lymphatic metastasis [120,121]. 

### 4.6. Tumor Invasion and Metastasis

Metastasis is a major cause of mortality in PDAC patients, and begins when tumor cells acquire the ability to invade and break through the basement membrane into the surrounding stroma, where they can then spread to distant organs such as the liver, lung, and peritoneum [122,123]. 

Macrophage-derived cytokines and chemokines including IL1β [124], CCL18 [125], and IL8 [126] can promote the epithelial-mesenchymal transition of pancreatic cancer cells through various signaling pathways, including PAR1 [127] and TLR4/IL10 activation [128]. Following co-culture with AAMs, pancreatic cancer cells demonstrate increased fibroblastic morphology, upregulated expression of mesenchymal markers Vimentin and Snail, alongside downregulation of the epithelial marker E-cadherin [128]. These changes are also associated with an increase in tumor cell proliferation, migration, and proteolytic activity [128]. In addition, TAMs secrete proteolytic enzymes, MMPs, and serine proteases, which are important components that mediate ECM degradation and cell-ECM interactions. For example, the macrophage pro-inflammatory chemokines CCL20 and MIP3α bind to CCR6 on the surface of PDAC cells to upregulate MMP9 expression and tumor invasion [41,42,43,44]. Macrophage-derived CCL18 also enhances the invasive ability of pancreatic cancer cells by inducing VCAM-1 expression. Reciprocally, VCAM-1-induced lactate production by pancreatic cancer cells polarizes macrophages towards an alternatively-activated endotype, thus forming a regulatory feedback loop within the tumor microenvironment [129]. Of note, TAMs that are associated with a pro-metastatic phenotype exhibit a pronounced glycolytic signature, and inhibition of glycolysis in TAMs disrupts their ability to support angiogenesis, tumor-cell extravasation, and EMT [40].

Primary tumors are capable of ‘priming’ distant organs and directing the mobilization of macrophages to these sites before tumor cells arrive [130]. These primed sites are referred to as pre-metastatic niches, and are largely established by macrophages that serve as a ‘landing guide’ for the homing of circulating tumor cells [131,132]. For example, the selective uptake of PDAC-derived exosomes by resident liver macrophages results in the activation of fibrotic pathways and the establishment of a pro-inflammatory milieu that supports metastasis [133]. Mechanistically, exosome-derived macrophage migration inhibitor factor (MIF) induces the release of TGFβ by resident liver macrophages, which in turn promotes the deposition of fibronectin by hepatic stellate cells [133]. These dense fibronectin deposits promote the accumulation of bone-marrow derived macrophages in the liver, and results in the formation of a pre-metastatic niche [133].

Consistent with the role of macrophages in supporting metastasis, pharmacological depletion of TAMs in mice reduces the spread of PDAC cells to the liver, lung, and spleen [20,39]. Strikingly, this anti-metastatic effect was independent of the presence of a primary tumor, since reduced lung metastasis was also detected in macrophage-depleted mice after tail vein injection of pancreatic cancer cells [39]. Likewise, in an orthotopic model of PDAC, immunosuppressive CCR2^+^ macrophages were recruited to the liver during tumor progression where they established a metastatic niche [21]. Once in the liver, macrophages secrete granulin which activate resident hepatic stellate cells into myofibroblasts that secrete periostin, resulting in a fibrotic microenvironment that sustains metastatic spread [85]. Disruption of the CCR2/CCL2 signaling axis or genetic depletion of granulin inhibits macrophage recruitment and protects against liver metastasis [21,85]. 

### 4.7. Drug Resistance/Modulating Treatment Response

One of the biggest clinical challenges of treating PDAC patients is the poor response of tumors to therapy, including radiotherapy and chemotherapy [134]. The ability of macrophages to modulate drug resistance and treatment response in PDAC has been extensively demonstrated using in vivo mouse models. The induction of EMT in pancreatic cancer cells by TAMs makes PDAC more resistant to chemotherapy [135]. Following treatment with radiotherapy or chemotherapy, PDAC tumor cells release inflammatory molecules including the chemokine CCL2, which recruits inflammatory macrophages to promote tumor proliferation and vascularization [18,19]. Tumor-derived CCL2 inhibits the efficacy of ablative radiotherapy and FOLFIRINOX chemotherapy in mice, while selective blockade of CCL2 using neutralizing antibodies blocked macrophage recruitment and restored the sensitivity of PDAC tumors to radiotherapy and chemotherapy [18,19]. TAMs can also mediate gemcitabine resistance by upregulating cytidine deaminase in tumor cells, an enzyme that metabolizes gemcitabine following its transport into the cell. Accordingly, inhibition of TAMs using a CSF1-receptor antagonist augmented the effect of chemotherapy in a gemcitabine-resistant PDAC mouse model [136]. In another study, TAMs were observed to rapidly metabolize gemcitabine in vitro, and pharmacological depletion of these cells using clodronate liposomes enhanced therapeutic response to gemcitabine in tumor-bearing mice [137]. TAMs can also directly support chemoresistance by secreting insulin-like growth factors (IGF)-1 and -2 which activate insulin/IGF receptors on pancreatic cancer cells [138], as well as Resistin, which binds to CAP-1 and TLR-4 on tumor cells and promotes gemcitabine resistance via STAT3 signaling [139].

## 5. Therapeutic Opportunities for Targeting TAMs in PDAC

**Figure 4:**
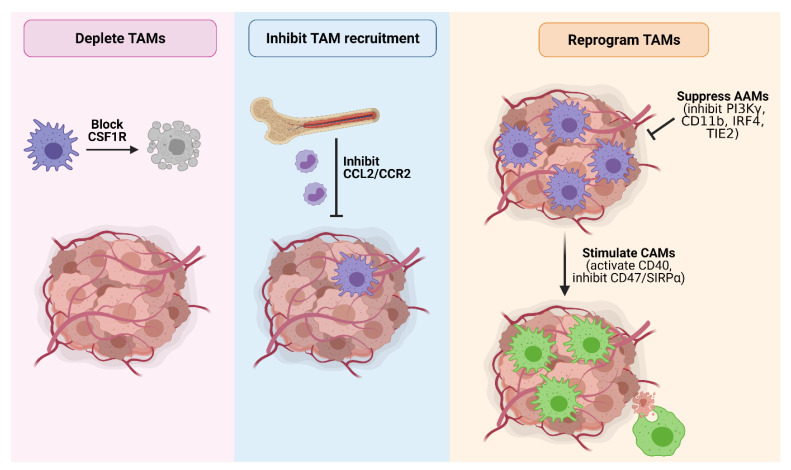
Strategies to therapeutically target TAMs in PDAC. Major TAM-targeting strategies in PDAC include (1) TAM depletion by blocking CSF1R signaling; (2) inhibition of TAM recruitment by blocking CCL2/CCR2 signaling; (3) suppressing the pro-tumorigenic activity of AAMs by inhibiting PI3Kγ, CD11b, IRF4 or TIE2; and (4) reprogramming TAMs towards a tumoricidal classically-activated endotype via CD40 agonists or inhibition of the ‘do not eat me’ CD47/SIRPα signaling axis to promote tumor cell phagocytosis. Figure created in Biorender.

Given the multi-faceted role of TAMs in promoting PDAC and their correlation with a poor prognosis, macrophages represent an attractive target to curb tumor development and progression. Indeed, TAM-targeting therapies have shown great promise in mouse models (Table 1), and a number of these agents are currently under clinical evaluation (Table 2). Major strategies targeting TAMs in PDAC include macrophage depletion, inhibiting macrophage recruitment, and macrophage reprogramming (Figure 4). 

### 5.1. Macrophage Depletion

Clodronate-mediated depletion of macrophages has been shown to improve T-cell infiltration [23], reduce metastasis [39,140], and enhance therapeutic response to chemotherapy [137] in pre-clinical mouse models of PDAC. However, this method is non-specific to TAMs and eliminates other CD11b^+^ phagocytotic cells in surrounding organs [39]. Meanwhile, the chemotherapeutic agent trabectedin activates caspase-8-dependent apoptosis in monocytes and macrophages, and results in the reactivation of tumor-infiltrating lymphocytes that secrete cytotoxic molecules to promote tumor cell death [141,142]. Lurbinectedin (PM00183) is an anti-cancer drug that triggers caspase-dependent apoptosis in cells by inducing DNA damage, and specifically depletes TAMs in the tumor microenvironment [143]. The combined use of lurbinectedin and gemcitabine induced a synergistic effect on PDAC xenografts by depleting TAMs, which led to cytidine deaminase downregulation in tumors and increased gemcitabine-mediated DNA damage [143].

Another method to deplete TAMs is by targeting CSF1R, which is expressed on the surface of circulating monocytes and tissue macrophages, and controls their survival, proliferation, differentiation, and chemotaxis [144]. CSF1R^+^ TAMs sustain PDAC growth through T-cell suppression, while targeted depletion of these cells using the selective CSF1R inhibitor AZD7507 enhances T-cell responses and upregulates the expression of immunogenic gene signatures [145]. In an orthotopic model of PDAC, macrophage depletion via CSF1R inhibition decreased the number of tumor-initiating cells, improved the efficacy of chemotherapy, inhibited metastasis, and unleashed T-cell cytotoxicity [20]. Meanwhile, remaining TAMs are reprogrammed by CSF1/CSF1R blockade to support antigen presentation and bolster T-cell activation, which further restrains tumor progression [146]. Depletion of TAMs by targeting CSF1 has also been reported to enhance the tumoricidal activity of radiation, in line with its association with promoting adaptive immunity [147]. Likewise, combining CSF1/CSF1R inhibitors with immunotherapies including anti-PD1 and anti-CTLA4 induces a synergistic effect and induces potent tumor regression in experimental models of PDAC [146]. Similarly, a triple combination of anti-PD1, an anti-CSF1R antibody, and a PDAC vaccine (GVAX) converted exhausted PD1^+^ T-cells to CD137^+^ activated effector T-cells, suggesting that adding a myeloid-targeting agent to vaccine-based cancer immunotherapy can reverse the anergy of T-cells in immune-quiescent tumors [148].

### 5.2. Blocking Macrophage Recruitment

Another strategy for targeting TAMs is by inhibiting their recruitment into the primary tumor. The CCL2/CCR2 signaling axis plays a central role in regulating the infiltration of circulating monocytes into the tumor microenvironment, making it a promising TAM-targeting therapy. In mice, CCR2 blockade prevents macrophage recruitment into the primary pancreatic tumor and pre-metastatic liver, and results in enhanced anti-tumor immunity, decreased tumor growth, and reduced metastasis [18,20,21]. A novel mechanism of radiotherapy resistance was recently discovered wherein PDAC cells respond to radiotherapy-induced stress by releasing high levels of CCL2 that recruit inflammatory monocytes to promote tumor proliferation and vascularity [19]. Accordingly, inhibition of inflammatory monocyte recruitment by antagonizing the CCL2/CCR2 chemokine axis in combination with ablative radiotherapy improved treatment response and enhanced survival [19]. 

A handful of inhibitors that selectively target CCL2/CCR2 signaling have completed Phase I clinical trials in pancreatic cancer (Table 2). An objective tumor response was seen in 49% of patients receiving FOLFIRINOX plus the CCR2 antagonist PF-04136309, with local tumor control achieved in 97% of patients (NCT01413022) [149]. In another trial, the CCR2 specific antagonist CCX872 was used in combination with FOLFIRINOX to treat subjects with locally advanced or metastatic non-resectable pancreatic cancer. Circulating monocytes, inflammatory monocytes, and myeloid-derived suppressor cells were reduced by CCX872-B plus FOLFIRINOX therapy, and resulted in an improved overall survival compared to chemotherapy alone (NCT02345408) [150]. 

### 5.3. Macrophage Reprogramming

Strategies aimed at reprogramming TAMs include inhibiting immunosuppressive AAMs, or by re-educating macrophages towards a tumoricidal CAM endotype. Here, we discuss known targets of TAM repolarization that have been investigated in PDAC models, and provide an overall summary of these targets in (Table 1). 

#### 5.3.1. PI3Kγ

PI3Kγ is a lipid kinase that promotes myeloid cell trafficking during inflammation and cancer [151], and drives the immunosuppressive transcriptional profile of TAMs [86]. In orthotopic and genetically-engineered mouse models of PDAC, inhibition of PI3Kγ slowed tumor growth, enhanced survival, and improved responsiveness to chemotherapy by inhibiting alternative macrophage polarization [86]. PI3Kγ depletion in TAMs significantly reduced the expression of immunosuppressive and pro-angiogenic genes, including *Arg1*, Tgfb, Il1b, Il6, and *Vegfa*. In contrast, the expression of immunostimulatory factors, including *Il12* and *Ifng*, was significantly enhanced in tumors and TAMs of PI3Kγ-deficient mice and PI3Kγ inhibitor-treated animals [86]. These findings were corroborated in another study, where PI3Kγ-mediated activation of the BTK signaling pathway in myeloid cells promoted the pro-tumorigenic activity of TAMs, while targeted inhibition of PI3Kγ or BTK in mice reprogrammed TAMs toward an inflammatory endotype that fostered CD8 T-cell cytotoxicity, suppressed PDAC growth, and improved responsiveness to chemotherapy [152,153]. 

#### 5.3.2. RIP1

Receptor-interacting protein 1 (RIP1) is an intracellular adaptor protein that relays signals from death receptors and TLRs to regulate inflammation, apoptosis, and necroptosis [154]. Pharmacologic inhibition of RIP1 reprograms TAMs towards a MHCII^hi^TNFα^+^IFNγ^+^ immunogenic phenotype in a STAT1-dependent manner, enhances cytotoxic T-cell activation, and impairs tumor growth in mice [155]. Notably, combined targeting of RIP1 and PD1 offers synergistic protection against primary PDAC and liver metastases, suggesting that RIP1 can be regarded as an immune ‘check-point kinase’.

#### 5.3.3. CD40

CD40 is a member of the TNFR superfamily that is constitutively expressed on all antigen-presenting cells (including TAMs and dendritic cells) and is critical for their activation and proliferation [156,157]. Binding of CD40 to its ligand CD40L, which is primarily expressed on the surface of activated T-cells, results in the upregulation of co-stimulatory molecules and proinflammatory cytokines that are crucial for T-cell priming [156,157]. In a spontaneous model of PDAC, administration of an agonistic anti-CD40 antibody led to the upregulation of MHC-II and CD86 on the surface of TAMs, and the production of pro-inflammatory cytokines including IL12, TNFα, and IFNγ. These CD40-activated macrophages rapidly infiltrated PDAC tumors and facilitated the depletion of the stroma [158]. In another study, a single dose of agonistic CD40 antibody in combination with gemcitabine and nab-paclitaxel enhanced TAM activation and the clonal expansion of T-cells that facilitated tumor destruction and durable remission [159]. The reprogramming of TAMs with anti-CD40 has also shown efficacy in increasing the intratumoral accumulation and longevity of TCR-engineered T-cells that promote tumor cell apoptosis [160]. Combining a T-cell-inducing vaccine with a PD1 antagonist and CD40 agonist antibody (triple therapy) induced T-cell priming and activation in mice with metastatic pancreatic cancer. The survival benefit was accompanied by a significant infiltration of IFNγ-, Granzyme B-, and TNFα-secreting effector T-cells, demonstrating that the combined use of CD40 agonist and PD1 antagonist antibodies can reprogram immune resistant tumors in favor of anti-tumor immunity [161].

Monoclonal CD40 agonistic antibodies (e.g., CP-870,893, APX005M, RO7009789 and SEA-CD40) have been evaluated in clinical trials. In a cohort of 21 patients with surgically incurable PDAC, administration of CP-870,893 in combination with gemcitabine resulted in a partial response in 4 out of 21 patients, and stable disease in 11 out of 21 of patients (NCT00711191) [158]. Strikingly, one patient with a partial response showed significant reduction of their primary tumor and complete resolution of two hepatic metastatic lesions. A second patient with a partial response underwent surgical resection of the primary tumor after achieving a complete resolution of all hepatic metastases and a 64% reduction in the primary tumor [158]. The tolerability and efficacy of the agonistic anti-CD40 antibody APX005M was also evaluated in combination with chemotherapy (gemcitabine and nab-paclitaxel), with or without Nivolumab in a Phase 1b study and showed clinical activity in patients with metastatic PDAC (NCT03214250) [162]. 

#### 5.3.4. CD11b

CD11b is an integrin molecule that is highly expressed on the surface of myeloid cells, and plays a key role in regulating their adhesion, chemotaxis, migration, phagocytosis, and survival [163,164]. Partial activation of CD11b using the small molecule agonist ADH-503 impaired tumor growth and improved survival in orthotopic and genetically-engineered mouse models of PDAC [165,166]. Mechanistically, ADH-503 decreased the number of immunosuppressive myeloid cells in the tumor and reprogrammed remaining TAMs towards a tumoricidal endotype with improved antigen-presenting properties [166]. Gene expression profiling of TAMs isolated from tumors of ADH-503-treated mice also revealed downregulated expression of immunosuppressive genes (e.g., *Il6*, *Tgfβ*, *Arg1*, *Il10*) and increased expression levels of the T-cell chemokine *Cxcl10*, which coincided with an increase in T-cell infiltration, activation, and proliferation [166]. Notably, CD11b agonism also synergized with anti-PD1 immune check-point blockade and promoted tumor regression, long-term survival, and lasting immunologic memory [166].

#### 5.3.5. CD47 and SIRPα

CD47 is a transmembrane protein expressed on normal cells, and acts as an anti-phagocytic ‘do not eat me’ signal. The binding of CD47 to signal regulatory protein α (SIRPα), which is mainly expressed by TAMs and dendritic cells, initiates a signaling cascade that prevents their phagocytic ability [167,168]. However, this mechanism is frequently hijacked by tumor cells that upregulate CD47, thereby enabling them to proliferate and avoid clearance by the immune system [169]. Furthermore, CD47-mediated protection against phagocytosis by macrophages prolongs the retention of exosomes in circulation, and enables cancer cells to manipulate their surroundings and support tumor growth [170]. Consequently, preventing CD47-SIRPα interactions removes this inhibitory check-point signal and enables the effective removal of cancer cells. Indeed, CD47 blockade using monoclonal antibodies facilitates the clearance of PDAC cells via macrophage-dependent phagocytosis in in vitro engulfment assays, and decreases metastatic tumor burden and improves survival in mice [140]. 

Single-cell RNA sequencing studies revealed that CD47 targeting induces compartmental remodeling of tumor-infiltrating immune cells within the PDAC tumor microenvironment by increasing the abundance of tumoricidal pro-inflammatory TAMs and reducing anti-inflammatory macrophages [171]. CD47 blockade also increased the number of intratumoral CD8 T-cells, and remodeled the T-cell cluster toward a more activated one [171]. Several microRNAs including miR340 and miR128 inversely correlate with CD47 expression in PDAC cancer cells and negatively regulate its expression [172,173]. Restoration of miR-340 expression in pancreatic cancer cells was sufficient to downregulate CD47 and promote the phagocytic ability of macrophages, while overexpression of miR-340 reprogrammed TAMs towards a classically-activated endotype and enhanced anti-tumor immunity by increasing CD8 T-cell recruitment [173]. Thus, targeting CD47-SIRPα interactions promotes the functional reprogramming of macrophages towards an activated tumoricidal endotype and augments macrophage-mediated clearance of cancer cells.

#### 5.3.6. IRF4

*IRF4* is a key transcription factor that regulates alternative-macrophage polarization [174], and suppresses the differentiation of CAMs [175]. Accordingly, IRF4 deficiency results in decreased expression of prototypical AAM-associated genes including *Arg1*, Ym1, and *Fizz1* [176]. In a syngeneic model of PDAC, inhibition of IRF4 via the immunomodulatory agent pomalidomide induced a shift from AAMs towards a pro-inflammatory population that decreased tumor fibrosis and promoted an immune-responsive environment with increased infiltration of activated T-cells [177]. Given that pomalidomide is FDA-approved and has also been shown to render pancreatic cancer cells susceptible to chemotherapy [178], these results highlight its potential as a combination therapy for the treatment of PDAC.

#### 5.3.7. TIE2

TIE2 is a receptor tyrosine kinase that identifies a lineage of proangiogenic monocytes required for tumor vessel formation, and is commonly used as a marker of alternatively-activated TAMs [179]. Of note, PDAC patients with a higher frequency of TIE2^+^ TAMs exhibit enhanced tumor neovascularization and a greater risk of developing metastatic disease [110,180]. The therapeutic benefit of targeting TIE2^+^ TAMs was demonstrated using the selective TIE2 inhibitor Rebastinib, which reduced pancreatic neuroendocrine tumor growth and metastasis in mice. Mechanistically, Rebastinib inhibited TIE2^+^ TAM infiltration and decreased the density of AAMs at the tumor invasive front, leading to impaired angiogenesis and tumor cell intravasation [180]. 

## 6. Challenges Associated with Targeting TAMs

Therapeutic modalities aimed at eliminating TAMs, inhibiting their infiltration, and/or reprogramming them towards a tumoricidal endotype have shown great potential in pre-clinical models. However, the effectiveness of these targeting strategies in preclinical models has not always translated into clinical trials [181]. This is in part due to the complexity associated with the nature and origin of TAMs (classically- versus alternatively-activated; locally-proliferating tissue-resident versus systemically-recruited macrophages) that co-exist within the tumor microenvironment at various stages of cancer progression. Furthermore, in vivo delivery barriers (e.g., low drug solubility, short half-life, and poor cellular uptake), as well as the immunosuppressive and desmoplastic PDAC tumor microenvironment dampen the efficacy of TAM-targeting agents. Given the role of macrophages in pathogen clearance and tissue homeostasis, the timing and duration of macrophage-targeting therapies also warrants further investigation to minimize the risk of side effects.

### 6.1. Differences between Mouse and Human Macrophages

Mouse models have been instrumental in elucidating the contribution of immune cells in tumor development and progression. However, there are fundamental differences in the development, activation, and function of mouse and human immune cells [182]. For example, the cell surface marker F4/80 is commonly used to identify mouse macrophages but is undetectable on human cells [183]. While CD68 is used as an alternative to distinguish human macrophages, it can also be expressed by some stromal and cancer epithelial cells [184]. Mouse and human monocyte-derived macrophages also exhibit differences in their response to IL4 and IL13 in vitro. Stimulation of mouse macrophages with IL4/IL13 promotes the upregulation of the prototypical-AAM genes *Ym1* and *Arg1*; however, this response is not observed in human macrophages [185]. Likewise, metabolism of the amino acid arginine by NOS2 and ARG1 is used to distinguish between CAMs and AAMs in mice, respectively, but this does not apply to human cells [29]. Thus, interspecies differences should be carefully considered to best translate our findings from pre-clinical models to human patients. 

### 6.2. Maximizing Translatability between Pre-Clinical Models and Human Patients

In many pre-clinical studies, TAM-targeted therapies commence when the primary tumor reaches a designated size (e.g., ~100 mm^3^ for subcutaneous tumor models). Treatment response is assessed by tumor shrinkage, impaired tumor growth, and/or Kaplan–Meier survival analysis. Although this method is a useful way of measuring the therapeutic efficacy of anti-cancer drugs in primary tumors, it does not reflect how most therapies are evaluated in clinical trials in which patients are enrolled with metastatic disease and with prior lines of failed treatment. Furthermore, many studies have shown that the tumor microenvironment at the primary site can dramatically differ from that of metastatic lesions [186,187]. While CSF1R inhibitors and CD40 agonists potently reduce tumor growth in mice, the combined use of Emactuzumab (anti-CSF1R monoclonal antibody) with Selicrelumab (agonistic CD40 monoclonal antibody) in patients with metastatic PDAC has not translated into objective clinical responses (NCT02760797) [188]. Likewise, CSF1R blockade and anti-PD1 therapy induces tumor regression in mice [146], but shows limited anti-tumor activity in PDAC patients with advanced disease (NCT02713529) [189]. To maximize the translational impact of pre-clinical studies, a complementary approach may include the use of models that mimic the stage of cancer in which the therapy is to be tested in clinical trials. One example to recapitulate advanced disease in mice is the intrasplenic pancreatic tumor model, in which syngeneic PDAC tumor cells are injected into the spleen and migrate into the liver via the portal vein, which is a common route of metastasis in human PDAC patients [190]. Likewise, the pre-clinical evaluation of TAM-targeting therapies in a neo-adjuvant setting or in combination with existing treatment paradigms to better emulate the design of clinical trials may also improve the chances of translational success.

### 6.3. Limited Markers to Discriminate between TAMs and Normal Macrophages

To maximize the translatability of pre-clinical studies, a thorough understanding of how TAMs evolve during malignancy, following therapy, and between primary and metastatic tumors is required for both mouse and human PDAC. Central to this is the identification of novel TAM targets that will allow the selective targeting of immunosuppressive or tumor-promoting endotypes, as there are currently limited markers to distinguish them from normal macrophages in non-tumor tissues. To this end, increased accessibility to novel technologies such as single-cell sequencing, spatial transcriptomics, and high-resolution imaging is anticipated to achieve a more comprehensive understanding of TAM subsets and their functional heterogeneity within the tumor microenvironment, as well as the identification of novel TAM targets to improve patient outcomes in PDAC [191].

### 6.4. Dissecting the Heterogeneity of Human PDAC Tumors

Compared to mouse PDAC tumors that are relatively homogenous, human PDAC tumors exhibit a high degree of heterogeneity between individuals (intertumoral heterogeneity), and within the same tumor (intratumor heterogeneity). Stratification models based on transcriptomic and genomic signatures have identified the existence of genetically-distinct PDAC subtypes with prognostic and biological relevance [192,193,194,195,196,197,198,199]. Both squamous/mesenchymal and immunogenic subtypes exhibit an enrichment of macrophage gene signatures compared to the pancreatic progenitor or aberrant differentiated endocrine exocrine (ADEX) subtypes, suggesting the differential contribution of macrophages in these tumors [194]. These findings were corroborated in another study demonstrating that CSF1R^+^ TAMs maintain key transcriptional signatures that define the squamous subtype of human PDAC [145]. Although one might speculate that TAM-targeting strategies may be more beneficial in subtypes with higher macrophage density, recent works using single-cell transcriptomics demonstrate that several subtypes may co-exist within a tumor [199,200]. Thus, targeting the immune cell compartment of PDAC without understanding its complexity could have deleterious or negligible impacts on clinical outcomes. 

### 6.5. Minimizing off-Target Side Effects

While pharmacologic depletion of TAMs has shown remarkable success in controlling PDAC in experimental models, its translational value in human patients remains limited. A major barrier to clinical translation is the availability of efficient and safe methods to deliver TAM-depleting agents into tumors following systemic administration. This is particularly important because systemic macrophage depletion in an immunocompromised patient undergoing chemotherapy may increase their vulnerability to infections, or result in impaired pathogen clearance. Tissue-resident macrophages are also important in maintaining homeostasis, and the prolonged depletion of these cells may impair organ function [201,202]. For example, transient ablation of alveolar macrophages impairs innate immune responses, and leads to severe influenza infection in mice [203]. Meanwhile, systemic macrophage depletion may disrupt immune balance by causing a compensatory influx of neutrophils that promote excessive inflammation [204]. Indeed, CSF1R inhibition has been reported to trigger an increase of granulocyte-specific chemokines produced by cancer-associated fibroblasts, resulting in an influx of granulocytes into tumors [205]. These adverse effects pose a challenge for the administration of CSF1R inhibitors, but may be overcome by combining anti-CSF1R drugs with other therapeutic methods. For example, combining inhibition of CXCR2 with CSF1R stopped the recruitment of neutrophil/granulocytes and improved the therapeutic efficacy of anti-PD1 immune check-point blockade [205]. Meanwhile, dual blockade of PI3kγ and CSF1R using a nano-micelle encapsulating the PI3Kγ inhibitor BEZ235 and CSF1R-siRNA reduced AAMs, increased the abundance of CAMs, and suppressed tumor infiltration of myeloid-derived suppressor cells in mice [206]. 

Although macrophage reprogramming represents a more sophisticated method than systemic depletion, one significant challenge is maintaining the delicate balance between the tumoricidal and immune-regulating functions of TAMs. The prolonged stimulation of CAMs could result in chronic inflammation and tissue damage, while loss of AAMs may result in impaired wound-healing. Therefore, targeted strategies that can selectively reprogram TAMs within the tumor microenvironment without affecting other macrophages in normal organs warrants further investigation. Indeed, recent advances in nanotechnology and biomedical engineering have given rise to novel drug formulations based on nanoparticles to overcome issues surrounding non-specificity and promote cell-specific uptake [207]. However, further research is required to comprehensively characterize cell surface markers and signaling pathways that can uniquely differentiate TAMs from normal macrophages in order to prevent undesired side effects.

## 7. Concluding Remarks

Given the multi-faceted role of TAMs in promoting PDAC and their correlation with a poor prognosis, macrophages represent an attractive therapeutic target to impair tumor development and progression. Strategies aimed at targeting TAMs in pre-clinical models include macrophage depletion, inhibiting their recruitment, or re-educating immunosuppressive TAMs towards a tumoricidal endotype. Many of these therapeutic agents also demonstrate complementary effects when combined with chemotherapy and immune check-point blockade, suggesting the additive benefit of targeting TAMs alongside other cell populations to enhance anti-tumor immunity. However, despite showing great potential in pre-clinical studies, the efficacy of these drugs has not always translated into clinical trials. This is in part due to the complexity associated with the nature and origin of TAMs, and the diverse heterogeneity of PDAC tumors in humans compared to mice. Further research is therefore required to comprehensively characterize TAM subsets within the tumor microenvironment, and identify novel TAM targets that will enable the selective targeting of tumor-promoting endotypes to improve patient outcomes in PDAC.

**Table 1:** Summary of selected targets of TAM inhibition in PDAC mouse models.

Pathway	Method	Effect	Refs.
**Macrophage depletion**	Administration of macrophage toxin GdCl_3_	Blocked acinar-to-ductal metaplasia and the formation of pancreatic intraepithelial neoplastic lesions.	[37,38]
	Administration of Trabectedin	Activated caspase-8-dependent apoptosis in TAMs, and resulted in the reactivation of tumor-infiltrating lymphocytes that promote tumor cell death.	[141,142]
	Administration of clodronate-liposomes	Reduced tumor formation and metastasis by enhancing T-cell infiltration. Also synergized with gemcitabine chemotherapy to elicit a potent anti-tumor response.	[23,39,137,140]
	Administration of Lurbinectedin (PM00183)	Activated caspase-dependent apoptosis in cells by inducing DNA damage.	[143]
	Inhibiting CSF1R	Decreased tumor-initiating cells, reduced immunosuppression, and improved chemotherapeutic responses.	[20]
		Reduced tumor burden due to increased tumor cell death and an enhanced T-cell immune response.	[145]
		Improved response to immune check-point blockade, resulting in tumor regression.	[146]
**Macrophage recruitment**	Inhibiting CCR2	Enhanced anti-tumor immunity, decreased tumor growth, and reduced metastasis.	[21]
		Decreased number of cancer stem cells in pancreatic tumors and improved response to chemotherapy.	[20]
		Suppressed radiation-induced neovascularization and enhanced the efficacy of radiotherapy.	[19]
**Macrophage reprogramming (suppressing AAM polarization)**	Inhibiting PI3Kγ	Inhibited tumor cell invasion, metastasis, and desmoplasia.	[86,151]
		Slowed tumor development, impeded late-stage tumor growth and improved responsiveness to chemotherapy.	[152,153]
	Inhibiting CD11b	Reduced expression levels of immunosuppressive genes in TAMs, and enhanced T-cell immunity.	[166]
	Inhibiting IRF4	Depleted pancreatic lesions of AAMs and generated an inflammatory and immune-responsive environment.	[177]
	Inhibiting Mstr1	Decreased tumor size, suppressed alternative macrophage polarization, and enhanced T cell infiltration.	[208]
	Administration of IL27	Inhibited M2 macrophages polarization, dampened the proliferation, migration, and metastasis of pancreatic cancer cells, and enhanced the efficacy of gemcitabine.	[209]
	Inhibition of TIE2	Reduced tumor angiogenesis and presence of alternatively-activated macrophages at the invasive tumor front.	[180]
**Macrophage reprogramming (stimulating CAM polarization)**	Inhibiting RIP1	Reprogrammed TAMs towards an MHCII^hi^TNFα^+^IFNγ^+^ immunogenic phenotype, and enhanced cytotoxic T-cell activation.	[155]
	Activating CD40	Improved infiltration of activated macrophages into tumors and depletion of tumor stroma. When combined with chemotherapy, anti-CD40 enhanced TAM activation and the clonal expansion of T-cells that resulted in tumor remission.	[158,159]
		Increased intratumoral accumulation and longevity of TCR-engineered T-cells that promote tumor cell apoptosis.	[160]
		When combined with a T-cell-inducing vaccine and anti-PD1 immune check-point blockade, anti-CD40 reprogrammed macrophages improved T-cell priming and activation.	[161]
	Blocking CD47	Improved macrophage-dependent phagocytosis of cancer cells, reprogrammed TAMs towards a pro-inflammatory tumoricidal endotype, and increased the number of intratumoral CD8 T-cells.	[140,171]

**Table 2:** Summary of selected clinical trials targeting TAMs in pancreatic cancer.

Class	Target	Compound/Class	Combination Partners	Phase	Sponsor	Trials ID	Status
**Small molecule**	CSF1R	Pexidartinib	Durvalumab (anti-PDL1)	I	AstraZeneca and Plexxikon	NCT02777710	Complete [210]
		ARRY-382	Pembrolizumab (anti-PD1)	II	Pfizer	NCT02880371	Complete [211]
	CCR2	PF-04136309	FOLFIRINOX	I	Washington University	NCT01413022	Complete [149]
		CCX872	FOLFIRINOX	I	ChemoCentryx	NCT02345408	Ongoing [150]
	VEGFR	Cediranib	Olaparib (PARP inhibitor)	II	AstraZeneca	NCT02498613	Ongoing, unpublished
	TGFβ	Galunisertib	Durvalumab (anti-PDL1)	I	AstraZeneca	NCT02734160	Complete [212]
	PI3K	Buparlisib (BKM120)	FOLFOX6	I	UNC Lineberger	NCT01571024	Complete, unpublished
		LY3023414	Gemcitabine/Capecitabine and Abemaciclib	II	Eli Lilly and Company	NCT02981342	Complete [213]
	BTK	Ibrutinib	Nab-paclitaxel and Gemcitabine	III	Pharmacyclics LLC	NCT02436668	Complete [214]
**Monoclonal antibody**	CSF1R	Emactuzumab (RG7155)	RG7876 (anti-CD40)	I	Hoffmann-La Roche	NCT02760797	Complete [188]
		AMG820	Pembrolizumab (anti-PD1)	I	Amgen	NCT02713529	Complete [189]
		Cabiralizumab (FPA008)	Nivolumab (anti-PD1)	I	FivePrime/BMS	NCT02526017	Complete [215]
		MCS110	PDR001 (anti-PD1)	I/II	Novartis	NCT02807844	Complete [216]
		Cabiralizumab	Nivolumab (anti-PD1) and Stereotactic Body Radiotherapy	II	NYU Langone Health	NCT03599362	Complete [217]
		Cabiralizumab	Nivolumab (anti-PD1), gemcitabine, Nab-paclitaxel	II	Bristol-Myers Squibb	NCT03336216	Ongoing[215]
		IMC-CS4 (LY3022855)	Cyclophosphamide, pembrolizumab (anti-PD1), GVAX	I	Merck Sharp & Dohme Corp.Eli Lilly and Company	NCT03153410	Ongoing, unpublished
	CD40	CP-870,893	Gemcitabine	I	Hoffmann-La Roche	NCT00711191	Complete [158]
		RO7009789	Nab-paclitaxel and Gemcitabine	I	Abramson Cancer Center of the University of Pennsylvania	NCT02588443	Complete, unpublished
		APX005M (Sotigalimab)	Nivolumab, Gemcitabine, Nab-Paclitaxel	I/II	Parker Institute for Cancer Immunotherapy	NCT03214250	Ongoing[162]
		SEA-CD40	Pembrolizumab, Gemcitabine, and Nab-Paclitaxel	I	Seagen Inc.	NCT02376699	Ongoing[218]

## Data Availability

Not applicable.
